# P-1352. Epidemiology and Clinical Profile of Infections Caused by Carbapenem-resistant Gram-negative Bacilli Identified by a Rapid Molecular Method in a Referral Hospital in Nicaragua

**DOI:** 10.1093/ofid/ofae631.1529

**Published:** 2025-01-29

**Authors:** Josué Zúniga-Gaitán, Sunaya Marenco-Avilés, Kevin Gavarrete-Rivas, Guillermo D Porras-Cortés

**Affiliations:** Hospital Dr. Fernando Vélez Paiz, Catarina, Masaya, Nicaragua; Hospital Dr. Fernando Vélez Paiz, Catarina, Masaya, Nicaragua; Hospital Dr. Fernando Vélez Paiz, Catarina, Masaya, Nicaragua; Hospital Dr. Fernando Vélez Paiz, Catarina, Masaya, Nicaragua

## Abstract

**Background:**

Bacterial resistance is a public health problem and resistance to carbapenems has a special connotation and it is important that each institution and country establish the characteristics of these infections. The objective of this study was to determine the epidemiology, clinical profile, and outcome of infections caused by carbapenem-resistant microorganisms in a referral hospital in Nicaragua.

**Figure 1.**
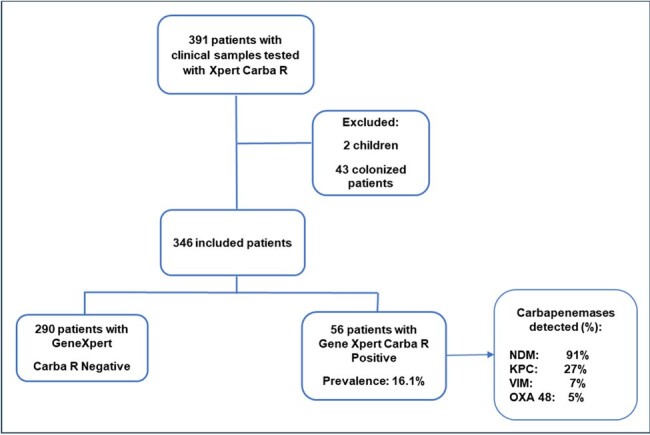
Prevalence of Carbapenemases Detected by Xpert Carba-R and Types of Carbapenemases in Clinical Samples

**Methods:**

An ambidirectional cohort study was carried out between January 2019 and November 2023 at the Dr. Fernando Vélez Paiz Hospital (Managua, Nicaragua) including all patients with infection and a clinical sample that had been processed directly by the Xpert Carba-R® rapid diagnostic molecular method to detect carbapenemases of the NDM, KPC, VIM, IMP, and OXA-48 type. Patients of the Surgery, Orthopedics, Internal Medicine, and Critical Care Department were included. The clinical data were obtained from the hospital's electronic medical record system.

**Figure 2.**
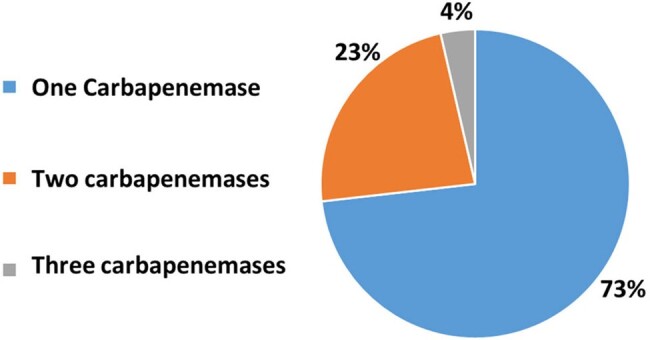
Number of Carbapenemases Detected by Patients

**Results:**

A total of 346 patients with infectious processes had their clinical samples processed directly by Xpert Carba-R®, and 56 (16.1%) had a carbapenem resistance gene detected (Figure 1). By type of infection, resistance to carbapenems was 36% in ventilator-associated pneumonia, 34% in intra-abdominal infection, and 30% in surgical site infection. NDM carbapenemase was present in 91.1% of patients, and KPC in 27% (Figure 1). More than one type of carbapenemase was found in 26.8% of patients. In 4% of the patients 3 types of carbapenemases was found. (Figure 2). The most frequently identified bacterium was *K. pneumoniae* (36.4%), followed by *E. coli* (16.9%) (Figure 3). Mortality in patients with carbapenem resistance was 58.9%. Risk factors for mortality were infection with the presence of two carpanemases, requiring mechanical ventilation on admission, presence of shock on admission, Charlson index ≥ 3 points, among others (Table 2).

**Figure 3.**
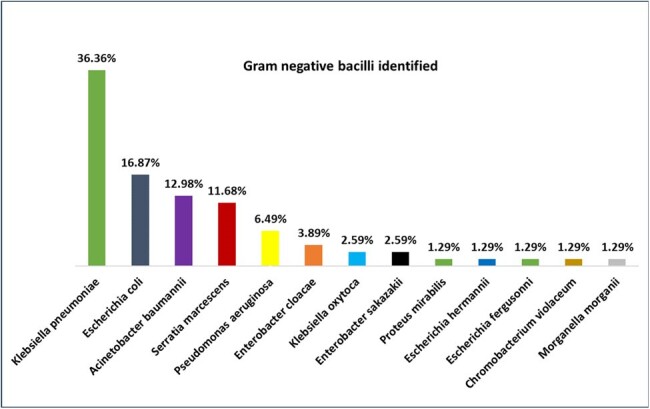
Gram Negative Bacilli Identified in Clinical Samples with Carbapenemases Detected by Xpert Carba-R

**Conclusion:**

The prevalence and mortality associated with the presence of carbapenemases detected by the molecular method used were high. The most prevalent carbapenemase was NDM. Different factors were associated with mortality, including shock on admission, invasive devices (eg: vascular catheter), patient comorbidities, and the presence of 2 different types of carbapenemases in the infection.

**Table 1.**
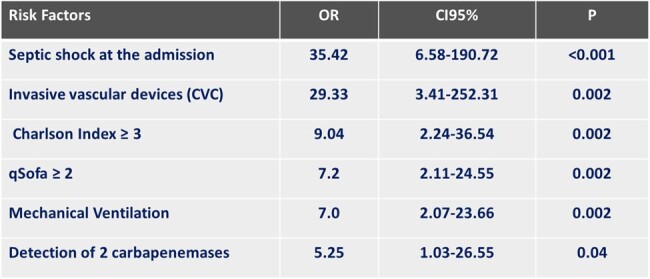
Risk Factors for Mortality in Patients with Infection with Gram Negative Bacilli Resistant to Carbapenems Detected by Xpert Carba-R

**Disclosures:**

**All Authors**: No reported disclosures

